# When the antidote for cyanide poisonings becomes a nightmare: an alarming outbreak of suicides using kits containing sodium nitrite

**DOI:** 10.1093/fsr/owad015

**Published:** 2023-07-04

**Authors:** Ricardo Jorge Dinis-Oliveira, Carlos Durão

**Affiliations:** TOXRUN – Toxicology Research Unit, University Institute of Health Sciences (IUCS), CESPU, 4585-116 Gandra, Portugal; Department of Public Health and Forensic Sciences, and Medical Education, Faculty of Medicine, University of Porto, 4200-319 Porto, Portugal; UCIBIO-REQUIMTE-Applied Molecular Biosciences Unit, Laboratory of Toxicology, Department of Biological Sciences, Faculty of Pharmacy, University of Porto, 4050-313 Porto, Portugal; Hospital Vila Franca de Xira, 2600-009 Vila Franca de Xira, Portugal; National Institute of Legal Medicine and Forensic Sciences, 1169-201 Lisbon, Portugal

**Dear editor,**

It was in 1936 that *Lancet* first published three cases of intoxication by nitrites [1]. The authors mentioned that poisoning by sodium nitrite was rare, and they were not able to trace any other record of fatal poisoning in the UK. Then, in 1939, three fatal cases due to sodium nitrite poisonings were reported in *Journal of the American Medical Association* (JAMA) [2]. That reality was maintained rare and virtually unheard of until very recently [3, 4]. Indeed, as forensic experts, we are very alarmed at how easily is being advertised and purchased this suicide means in different countries that ended in several series of fatal events [5–7]. In other words, more fatal cases of intoxication by sodium nitrite were published in the last 2 years than before. The toxicovigilance data justify urgent and restrictive regulations for the use of sodium nitrite, but perhaps the market around this substance restraints further measures to be undertaken. Indeed, since sodium nitrite has several industrial applications, accidental exposure to contaminated water and food but especially intentional ingestion has been associated with severe toxicity and deaths. Moreover, since routine tests for this substance are rarely available in different clinical and forensic settings, several other cases are certainly being missed. The tip of the iceberg as we unfortunately anticipated is becoming reality [7].

However, how people were aware of this possible means of suicide? Who is advertising? What kind of websites and services are helping the “exit”? We tried to obtain some answers, which were compiled here to alert clinical and forensic practitioners and toxicologists to this new trend of poisoning: (i) the networks supporting euthanasia provide receipts, step-by-step instructions, and recommendations on how to use exit suicide kits containing sodium nitrite. Some websites demonstrate a high number of views. For instance, the Sanctioned Suicide website drew over 10 million page views in September 2022 and as of March 2023, the forum has over 30 000 members; (ii) sodium nitrite also gained attractiveness through online pro-suicide forums probably due to efficacy and easy access; (iii) sodium nitrite is advertised as a peaceful suicide means due to its mechanism of toxicity; and (iv) this food additive for meat curing can be easily purchased with a high level of purity (98%–99%) via different online vendors and consumer marketplaces. For instance, Amazon, already in 2022 and 2023, has been accused in multiple lawsuits of selling “suicide kits” to vulnerable teens and young adults.

The taboo cannot be further ignored. Nevertheless, since the food industry uses sodium nitrite for legitimate and legal applications, it will be a major challenge to restrict and find real solutions for sodium nitrite worldwide access. UK has deemed sodium nitrite a “reportable substance”, meaning that retailers must report suspicious purchases of the compound by individuals to local authorities. Very recently in May 2023, a man was charged due to alleged online sales and distribution of sodium nitrite in the Greater Toronto Area and ultimately aiding the suicide [8]. This commentary highlights the increased probability of encountering unexplained cyanosis and methemoglobinemia in clinical and forensic intoxications. Moreover, authorities should be informed about suspicious purchases.
